# Revealing the biodiversity of Chilean birds through the COI barcode approach

**DOI:** 10.3897/zookeys.1016.51866

**Published:** 2021-02-11

**Authors:** Nelson Colihueque, Alberto Gantz, Margarita Parraguez

**Affiliations:** 1 Laboratorio de Biología Molecular y Citogenética, Departamento de Ciencias Biológicas y Biodiversidad, Universidad de Los Lagos, Avenida Alcalde Fuchslocher 1305, Casilla 933, Osorno, Chile Universidad de Los Lagos Osorno Chile; 2 Laboratorio de Ecología, Departamento de Ciencias Biológicas y Biodiversidad, Universidad de Los Lagos, Osorno, Chile Universidad de Los Lagos Osorno Chile; 3 Laboratorio de Genética, Acuicultura y Biodiversidad, Universidad de Los Lagos, Osorno, Chile Universidad de Los Lagos Osorno Chile

**Keywords:** Aves, biodiversity, COI, genetic variation, Neotropical birds, taxonomy

## Abstract

The mitochondrial cytochrome c oxidase subunit I (COI) gene is an effective molecular tool for the estimation of genetic variation and the identification of bird species. This molecular marker is used to differentiate among Chilean bird species by analyzing barcodes for 76 species (197 individuals), comprising 28 species with no previous barcode data and 48 species with sequences retrieved from the BOLD and GenBank databases. The DNA barcodes correctly identified 94.7% of the species analyzed (72 of 76 species). Mean intraspecific K2P distance was 0.3% (range 0–8.7%). Within the intraspecific divergence range, three species, *Phrygilus gayi*, *Sephanoides sephanoides* and *Curaeus curaeus*, showed relatively high intraspecific divergence (1.5–8.7%), possibly due to the presence of a species complex or geographic isolation of sub-populations. Mean interspecific K2P distance was 24.7% (range 1.3–43.5%). Consequently, the intraspecific K2P distance showed limited overlap with interspecific K2P distance. The mean intraspecific divergence in our study was similar to that found in temperate regions of South America (0.24%). However, it was approximately one order of magnitude lower than values reported for bird species in tropical regions of northern South America (1.8–2.13%). This result suggests that bird species from Chile show low levels of genetic structure and divergence. The small overlap between intra- and inter-specific distances implies that COI barcodes could be used as an effective tool to identify nearly all the Chilean bird species analyzed.

## Introduction

Birds are among the animal groups that have been subjected to extensive DNA barcoding. Currently, DNA barcodes are publicly available for 41% of known bird species in the world, with data for nearly 4300 species from 37 of 39 recognized avian orders (Barreira et al. 2016). The reported accuracy of species-level DNA barcoding is 93%–99% for birds, confirming its efficacy in discriminating among avian species and its potential as a tool for assigning unknown avian individuals to a species. The accuracy of this method in birds is attributable to fact that the maximum intraspecific distance is typically smaller than the minimum interspecific distance in these species. This so-called barcode gap (Meyer and Paulay 2005) means that the COI gene has the power to delineate species boundaries.

In recent years, a number of DNA barcoding studies have assessed the efficacy of using COI data to identify South American birds. Analyses of hundreds of bird species from countries in this region, such as Argentina (Kerr et al. 2009), Brazil (Chaves et al. 2015), and Ecuador and French Guiana (Milá et al. 2012), have shown that COI sequences are highly accurate for species-level identification (93–98%). Findings for birds from other geographic areas show similar levels of accuracy (Barreira et al. 2016). Moreover, these studies reveal deep intraspecific genetic divergence in Neotropical birds, likely associated with a more complex pattern of regional divergence than in the North American avifauna (Tavares et al. 2011; Milá et al. 2012). Thus, in some birds, intraspecific differences overlap with interspecies differences, especially in populations that include multiple sub-species or in samples from large geographic areas, ecoregions, or areas of endemism (Tavares et al. 2011). Therefore, the current taxonomy likely underestimates the biodiversity of the Neotropical avifauna. Given the complex genetic divergence patterns reported in this region, likely associated with a high incidence of non-monophyletic species, the general utility of DNA barcoding across different biogeographic regions of South America merits further attention.

The Chilean avifauna comprises of 443 species, if we only consider species that are residents or regular visitors (Couve et al. 2016), or those that meet the criteria of at least five records in the national territory (Martínez-Piña and González-Cifuentes 2017). These birds belong to 65 families distributed across broad altitudinal (0–6000 m) and latitudinal (18°S–56°S) gradients in various ecoregions of the country. This avifauna represents around 13% of all Neotropical bird species, estimated at 3370 species (Newton 2003). The Chilean avifauna is characterized by a fusion of South American relic elements (e.g., *Pygarrhichas* and Furnariidae) of Cenozoic origin with North American elements (e.g., members of the genus *Phrygilus*) that arose during the Pleistocene (Díaz 2005; González and Wink 2008; Álvarez-Varas et al. 2015). During this period, glacial cycles profoundly affected species distribution and gene flow patterns in populations throughout the world (Shepard and Burbrink 2009). Some studies have shown that Pleistocene climate fluctuations may have altered the distributions, sizes, and genetic structures of avian populations (Lovette 2005; van Els et al. 2012; Lougheed et al. 2013). Most of the elements that compose the Chilean avifauna originated during the Pleistocene, due to population differentiation events that occurred in paleorefuges in the Altiplano, central Chile, Patagonian temperate forests, and other areas (Cracraft 1985; Vuilleumier 1991; Díaz 2005; Masselo et al. 2011). These geological processes, along with the geographic isolation of Chile imposed by geographic barriers such as the Andes, the Pacific Ocean, and the northern desert, resulted in depleted species richness and a high level of endemism in relation to the size of this geographic area (Kelt et al. 2016). For example, around of 2.3% of terrestrial avifauna of Chile are endemic species (Kelt et al. 2016) that are largely restricted to certain ecoregions such as the Atacama Desert, Mediterranean forests, Valdivian temperate rain forests, Patagonian steppe, and dry Puna (Jaramillo 2014). Of note is that natural selection and local adaptation mechanisms associated with this geographic isolation seem to have also played an important role in the diversification of Chilean avifauna along the Andes mountains (Campagna et al. 2011; Álvarez-Varas et al. 2015).

Little is known about the population structure and genetic diversity of the taxa that currently compose the diversity of birds in Chile (Colihueque and Gantz 2019). Recent molecular studies of Chilean birds using COI sequences have provided insight into phylogenetic, phylogeographic and taxonomic issues (Masello et al. 2011; González 2014; Álvarez-Varas et al. 2015; Colihueque et al. 2015). This molecular tool also offers the opportunity for an in-depth evaluation of the genetic differentiation within and between Chilean bird species and to test its species-level resolution for bird identification. The particular evolutionary history of Chilean birds, associated with glacial events and isolation imposed by strong geographic barriers, has likely affected the gene flow and genetic variability of many species. Therefore, this approach may provide clues that would clarify, for example, the taxonomic status of various species, divergence patterns across different ecoregions, and the speciation process in Chile. Here, we examine the pattern of barcode divergence in a significant proportion of Chilean bird species, based on new COI sequences and sequences previously published in the Barcode of Life Data Systems (BOLD) (http://www.barcodinglife.org/) and GenBank databases.

## Materials and methods

### Sampling

We obtained samples from across central and southern Chile, mainly from the Cachapoal (34°S) and Osorno, Ranco, and Valdivia provinces (40°–41°S). Samples corresponded to dead birds found along the highways which were collected between 2012 and 2019 by volunteers and the authors; date and site of collection (at least at province level) were recorded immediately. Collection sites were georeferenced using Google Earth based on locality names. Information about vouchers and collection sites is available in Suppl. material 2: Table S1. The author A. Gantz, given his broad expertise in ornithology, performed the species identification according to standard diagnostic criteria based on morphology and feather coloration. Identification guides of Chilean birds (Jaramillo 2014; Couve et al. 2016) were also used to assist in the identification task. After identification, the specimens were photographed, and deposited in the bird collection of the Laboratorio de Biología Molecular y Citogenética of the Universidad de Los Lagos (ULA), Osorno, Región de Los Lagos, under identification numbers 1160ULA, 1161ULA, 1163ULA, 1164ULA, 1165ULA, 1167ULA, 1194ULA, 1195ULA, 1200ULA, 1201ULA, 1214ULA–1217ULA, 1235ULA–1237ULA, 1245ULA, 1249ULA, 1277ULA, 1278ULA, 1280ULA–1283ULA, 1295ULA, 1309ULA–1314ULA, 1316ULA, 1318ULA, 1320ULA, 1329ULA, 1332ULA–1334ULA, 1338ULA, 1339ULA, 1346ULA, 1347ULA, 1350ULA, 1354ULA, 1385ULA, 1388ULA, 1389ULA, 1391ULA, 1392ULA, 1395ULA, 1397ULA, 1400ULA, 1401ULA, 1403ULA, 1405ULA, 1460ULA, 1465ULA–1473ULA,1506ULA and 1507ULA (Suppl. material 2: Table S1). These specimens are publicly available for further investigation. A set of photographs of representative analyzed specimens is provided in Suppl. material 1. Tissue samples were taken mainly from the pectoral and femoral muscles as the size of these muscles facilitates dissection. Tissue samples were then fixed in 80% ethanol. DNA was extracted from the fixed muscle tissue using the phenol-chloroform method, as described in Taggart et al. (1992). Extracts were standardized at 100 ng/μL using Tris-EDTA buffer, pH 8.0. To obtain COI sequences of birds from Chile, we searched the BOLD Public Data Portal using the search terms [Aves Chile]. Subsequently, the recovered records were assessed for correct collection site (i.e., Collected in: Chile) and also by checking the correct geographic coordinates within Chile using Google Earth. The same verification process was used for COI sequences recovered from GenBank. The addition of these sequences enhances assessment of COI gene variability at the intraspecific and interspecific levels. Based on the location of collection sites, some species cover a wide latitudinal range across Chile. For example, *Thinocorus orbignyianus* covers a range from Tarapaca (20°S) to Santiago (33°S) (ca. 1700 km), and *Vanellus chilensis* from Santiago (33°S) to Magallanes (53°S) (ca. 2200 km). Species nomenclature follows the Clements et al. (2016) taxonomy. We analyzed 116 individuals from 42 species. In total we obtained sequences from 68 individuals of 32 species, including 28 species not previously barcoded. Lists of the newly sequenced specimens with those from BOLD and GenBank, as well as information about vouchers and collection sites, are available in Suppl. materials 2 (Table S1) and 3 (Table S2), respectively.

### PCR and sequencing

The primer pairs BirdF1(5’-TTCTCCAACCACAAAGACATTGGCAC-3’)and BirdR1(5’-ACGTGGGAGATAATTCCAAATCCTG-3’),as well as BirdF1 and BirdR2(5’-ACTACATGTGAGATGATTCCGAATCCAG-3’)(Hebert et al. 2004), were used for COI amplification. Resulting amplicons had a length of ca. 700 bp. When PCR failed, possibly due to degraded DNA, alternative M13-tailed primer pairs were used, following Ivanova et al. (2007): FishF2_t1 (5’-GTAAAACGACGGCCAGTCGACTAATCATAAAGATATCGGCAC-3’) and FishR2_t1 (5’-CAGGAAACAGCTATGACACTTCAGGGTGACCGAAGAATCAGAA-3’). In addition, when FishF2_t1 and FishR2_t1 also failed the internal primer pairs AvMiF1 (5’-CCCCCGACATAGCATTCC-3’) and AvMiR1 (5’-ACTGAAGCTCCGGCATGGGC-3’) in conjunction with BirdF1 and BirdR1 were used. PCR amplification was carried out in 15 μL using a reaction mix composed of 3 μL Taq polymerase buffer (1×), 2 μL of enhancer (0.7×), 0.3 μL of dNTPs (0.2 mM), 0.45 μL of MgCl_2_ (1.5 mM), 0.3 μL of each primer (0.2 μM), 0.06 μL of Taq DNA polymerase (0.02 U/ μL) (Kapa Biosystems), 3 μL of template DNA (20 ng/μL), and 5.59 μL of DNAse/RNAse free distilled water (Gibco). Thermal cycling was performed as follows: initial denaturation at 94 °C for 2 min followed by 40 cycles of 94 °C for 45 s, annealing temperature of 52 or 58 °C, depending on the primer pairs, for 45 s, 72 °C for 45 s, and a final extension step at 72 °C for 5 min. PCR products were visualized on 2% agarose gels and cleaned prior to sequencing with an QIA quick gel extraction kit (Qiagen). PCR products were bi-directionally sequenced on an Applied Biosystems ABI377 automated sequencer. Sequence records were assembled from forward and reverse reads using GENEIOUS 4.0.2 software (Biomatters Ltd.). We checked for potential amplifications of pseudogenes (NUMTs) by translating the COI sequence into amino acid sequences using mitochondrial vertebrate genetic code. When unexpected stop codons, frameshifts, or unusual amino acidic substitutions were observed, the sequence was discarded from the analysis. All sequences were deposited in GenBank (accession numbers MG263831 to MG263870 and MN986932 to MN986959).

### Data analyses

The sequences obtained were aligned and edited using GENEIOUS 4.0.2 software (Biomatters Ltd.). Base substitution saturation, a phenomenon that may decreases the amount of phylogenetic information contained in a sequence dataset, was tested based on the index of substitution saturation (ISS) (Xia et al. 2003), which assumes a critical index of substitution saturation (ISSc) that defines a threshold for significant saturation in the data. This analysis was performed using DAMBE v. 5.3.105 (Xia 2013). For all sequence comparisons, the Kimura 2-parameter distance model (K2P) (Nei and Kumar 2000) was used. This metric was chosen because its performance for species identification is equivalent to other models (Collins et al. 2012). This genetic distance was calculated using MEGA 5.05 software (Tamura et al. 2011). Pairwise sequence divergence was calculated separately for intraspecific and interspecific distances as well as for intrageneric comparisons. Mean intraspecific distances were calculated for species that were represented by at least two specimens. Intrageneric K2P genetic distances were calculated based on at least two species for a particular genus. The best-fit nucleotide substitution model was determined using jModelTest 2.1. (Darriba et al. 2012) based on Bayesian Information Criterion (BIC). The best model was then used with maximum likelihood (ML) analyses to construct a ML tree. The consistency of topologies (nodal support) was estimated using a bootstrap approach with 1000 bootstrap replications (Felsenstein 1985). Phylogenetic trees were rooted with one representative of Great Tinamu (*Tinamus major*). To include all sequences developed in this study and as many as sequences as possible from BOLD and GenBank, we used 443-bp sequence length alignments. A species was considered distinguishable by DNA barcode if: a) it was monophyletic (i.e., the species formed a single cluster) and b) it did not share a barcode with any other species.

## Results

### Sequences dataset and model of nucleotide substitution

Barcodes were analyzed for a total of 76 unique species (197 individuals), including 32 species sequenced in this study (68 individuals) and 48 species (129 individuals) from BOLD and GenBank (see Suppl. material 2, 3 – respectively). These sequences represent 17.2% (76/443) of known species from Chile. This dataset comprised 36 families. The COI sequences of new specimens ranged in size from 464 to 750 bp, with a mean length of 655.3 bp. The average number of sequences per species was 2.6 (range 1–17), with 43 species (56.6%) presenting between 2 and 17 sequences. After aligning the 197 sequences, a dataset of 443-pb sequence length were obtained. For this alignment, 193 variable positions and 182 parsimony-informative sites were found. Overall nucleotide frequencies were: A (24.7%), T (26.5%), C (33.3%) and G (15.4%). The best fit-model of nucleotide substitution was Hasegawa-Kishino-Yano model (HKY) with a fraction of invariable sites and gamma distribution (HKY+I+G) (BIC value = 22738.5785). No evidence of base saturation was found, since the ISS value (0.219) was significantly lower (T = 15.4, d.f. = 443, P < 0.0001) than the observed ISS.c value (0.696).

### Species identification

**Figure 1.**
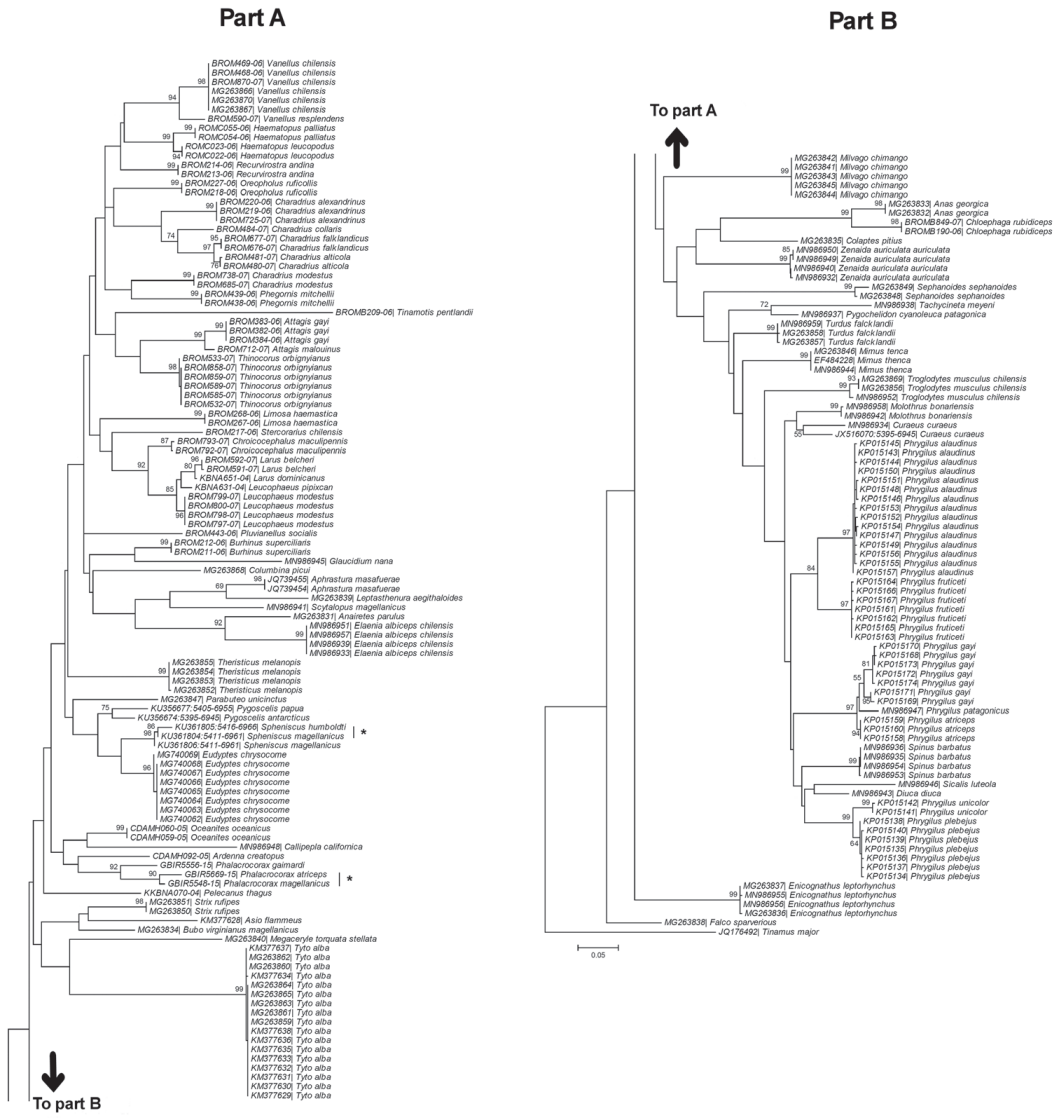
Maximum likelihood (ML) tree derived from the analysis of COI sequences for 76 bird species from Chile. The numbers at the nodes represent the percentage of bootstrap support. GenBank or BOLD accession number for each specimen are shown. Scale indicates the sequence divergence estimated from the number of nucleotide substitutions per site. The asterisk indicates the contradictory clusters found in the tree. *Charadrius alexandrinus* is currently known as *Charadrius nivosus*.

The COI barcode correctly identified 94.7% of the species studied (72 of 76 species). That is, these 72 species had unique DNA barcodes that did not overlap with the barcodes of any other species. Interspecific K2P distance ranged from 1.3 to 43.5% (mean 24.7%). In most cases, the ML tree, as shown in Figure 1, reflected a relatively low within-species divergence as compared to between-species divergence. Most of the terminal groups included specimens of the same species in a single cluster, as expected for samples of the same species in a monophyletic group. Bootstrap values were above 80, except for *Curaeus curaeus*, *Phrygilus gayi* and *Phrygilus plebejus* (Figure 1). The phylogenetic analysis showed a close phylogenetic relationship in the *Phalacrocorax* and *Spheniscus* genera, i.e., some species clustered together within the same branch. For example, *P. atriceps* clustered together with *P. magellanicus* and *S. humboldti* grouped in the same cluster with *S. magellanicus*. In addition, these species pairs exhibited a very similar COI barcode given that the K2P distance was relatively low (3.8% and 2.2%, respectively). These clusters were relatively well-supported (86–90% bootstrap support), we considered that this result may suggest the occurrence of reciprocal non-monophyly. However, this conclusion should be treated with caution due to the low number of COI sequences analyzed and, therefore, further analysis of additional samples will be necessary to support this result.

**Table 1.** Comparisons of K2P-pairwise distances at two taxonomic levels for 76 species of birds from Chile. Intraspecific distance was calculated for species which two or more sequences were available.

Taxonomic level	Number of individuals	Number of taxa	Number of comparisons	Genetic distances (%)			
				Mean	SE	Minimum	Maximum
Intraspecific	164	43	448	0.3	0.0	0.0	8.7
Interspecific	197	76	2850	24.7	0.1	1.3	43.5

**Figure 2.**
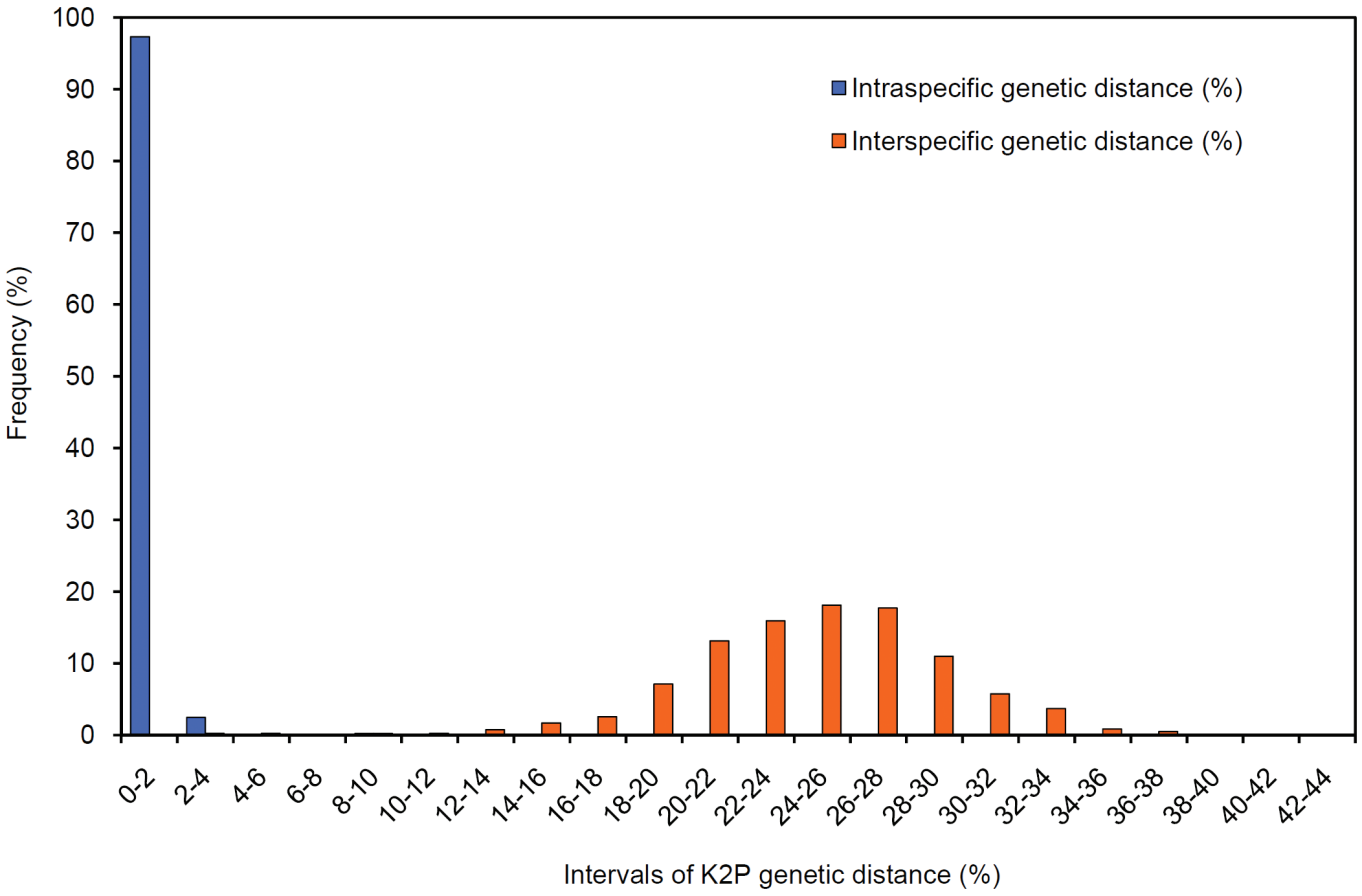
Histogram showing the distribution of K2P-pairwise genetic distances based on the COI gene of 76 bird species from Chile. Intraspecific distances are indicated with blue bars and interspecific distances, excluding within-species comparisons, are shown with red bars.

### Intra- and interspecific divergence

**Figure 3.**
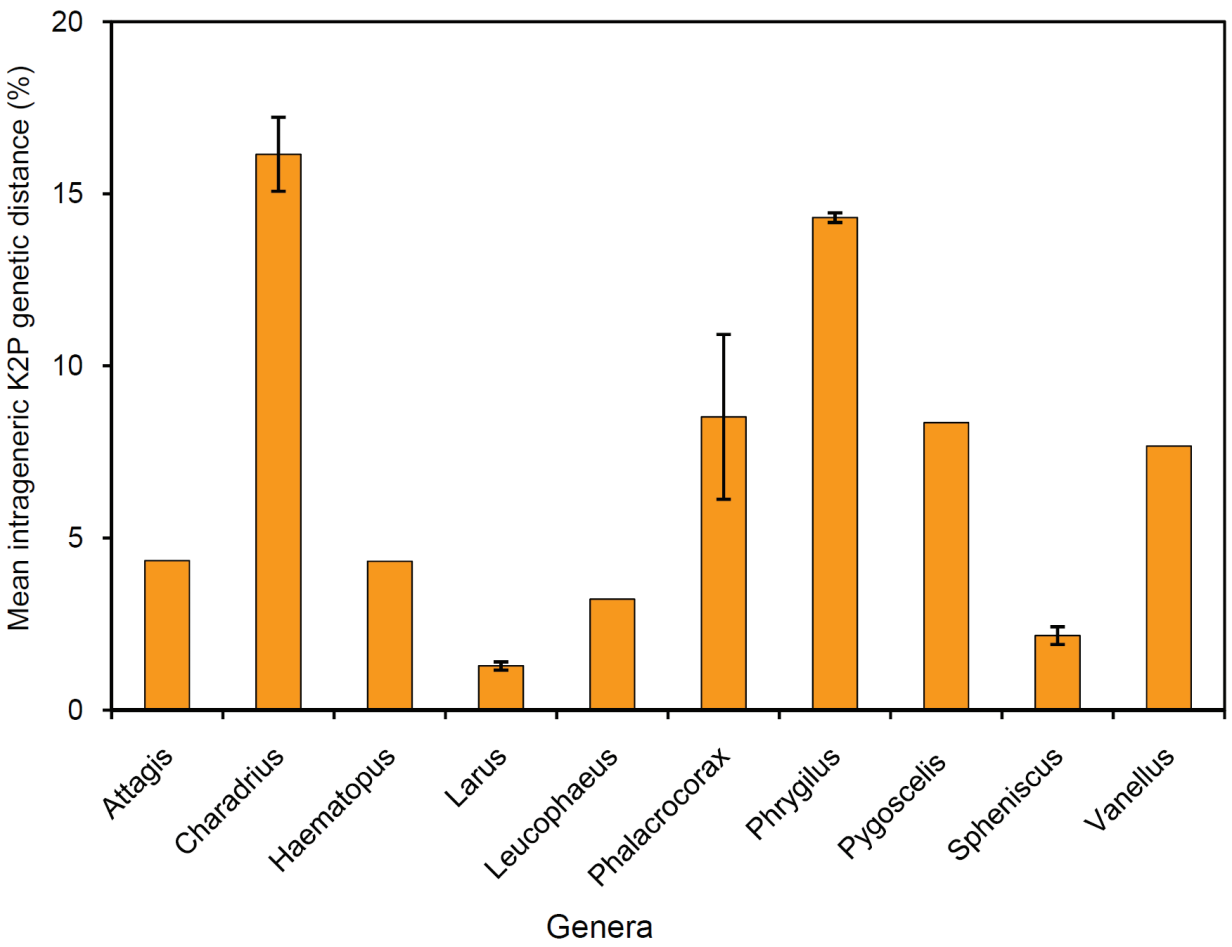
Mean intrageneric K2P genetic distance of ten genera of birds from Chile. Error bars represent the standard error of the mean.

The between-species differences in COI sequences were greater than the within-species differences. Mean intraspecific K2P distance was 0.3% (range 0–8.7%), while mean interspecific K2P distance was ca. two orders of magnitude larger, at 24.7% (range 1.3–43.5%) (see Table 1). Consequently, the intraspecific K2P distance showed limited overlap with interspecific K2P distance (Figure 2). In fact, only 22 of 2850 species-pair comparisons (0.77%) showed a divergence level below the maximum intraspecific K2P distance (8.7%), such as were recorded for *Larus dominicanus* and *Larus belcheri* (1.3%), *Charadrius falklandicus* and *Charadrius alticola* (2.1%), and *Phrygilus atriceps* and *Phrygilus gayi* (2.9%). Three species had a relatively high intraspecific divergence that overlapped with the minimum interspecific distance, namely, *Curaeus curaeus* (mean K2P = 8.7%), *Phrygilus gayi* (mean K2P = 1.5%) and *Sephanoides sephanoides* (mean K2P = 2.2%) (Table 2). Mean intrageneric K2P distance varied widely, from 1.3 to 16.1% (see Table 3 and Figure 3). This distance was low in *Larus* (1.3%), *Spheniscus* (2.2%), *Leucophaeus* (3.2%), *Attagis* (4.3%) and *Haematopus* (4.3%) genera, reflecting a pattern of low genetic divergence among these closely related species. The greatest intrageneric divergence levels were observed in *Charadrius* (16.1%) and *Phrygilus* (14.3%).

**Table 2.** Intraspecific K2P genetic distances for 43 species of birds from Chile, calculated when two or more sequences were available. Bold face indicates species with high mean intraspecific divergence that overlapped the minimum interspecific distance (> 1.3%). ‡ Currently known as *Charadrius nivosus*.

Species or subspecies	Number of individuals	Genetic distances (%)			
		Mean	SE	Minimum	Maximum
*Anas georgica*	2	0.0	0.0	0.0	0.0
*Aphrastura masafuerae*	2	0.0	0.0	0.0	0.0
*Attagis gayi*	3	0.0	0.0	0.0	0.0
*Burhinus supercialiaris*	2	0.0	0.0	0.0	0.0
*Charadrius alexandrinus*‡	3	0.0	0.0	0.0	0.0
*Charadrius alticola*	2	0.2	0.2	0.2	0.2
*Charadrius falklandicus*	2	0.0	0.0	0.0	0.0
*Charadrius modestus*	2	0.2	0.2	0.2	0.2
*Chloephaga rubidiceps*	2	0.0	0.0	0.0	0.0
*Chroicocephalus maculipennis*	2	0.5	0.3	0.5	0.5
***Curaeus curaeus***	**2**	**8.7**	**1.7**	**8.7**	**8.7**
*Elaenia albiceps chilensis*	4	0.0	0.0	0.0	0.0
*Enicognathus leptorhynchus*	4	0.2	0.2	0.0	0.5
*Eudyptes chrysocome*	8	0.1	0.1	0.0	0.5
*Haematopus leucopodus*	2	0.0	0.0	0.0	0.0
*Haematopus palliatus*	2	0.0	0.0	0.0	0.0
*Larus belcheri*	2	0.2	0.2	0.2	0.2
*Leucophaeus modestus*	4	0.0	0.0	0.0	0.0
*Limosa haemastica*	2	0.0	0.0	0.0	0.0
*Milvago chimango*	5	0.0	0.0	0.0	0.0
*Mimus thenca*	3	0.0	0.0	0.0	0.0
*Molothrus bonariensis*	2	0.2	0.2	0.2	0.2
*Oceanites oceanicus*	2	0.0	0.0	0.0	0.0
*Oreopholus ruficollis*	2	0.0	0.0	0.0	0.0
*Phegornis mitchelli*	2	0.0	0.0	0.0	0.0
*Phrygilus alaudinus*	15	0.5	0.1	0.0	0.9
*Phrygilus atriceps*	3	0.0	0.0	0.0	0.0
*Phrygilus fruticeti*	7	0.3	0.1	0.0	0.5
***Phrygilus gayi***	**7**	**1.5**	**0.4**	**0.2**	**3.2**
*Phrygilus plebejus*	7	0.3	0.1	0.0	0.5
*Phrygilus unicolor*	2	0.2	0.2	0.2	0.2
*Recurvirostra andina*	2	0.0	0.0	0.0	0.0
***Sephanoides sephanoides***	**2**	**2.2**	**0.7**	**2.2**	**2.2**
*Spheniscus magellanicus*	2	0.5	0.3	0.5	0.5
*Spinus barbatus*	4	0.1	0.1	0.0	0.2
*Strix rufipes*	2	0.0	0.0	0.0	0.0
*Theristicus melanopis*	4	0.1	0.1	0.0	0.2
*Thinocorus orbignyianus*	6	0.1	0.1	0.00	0.2
*Troglodytes musculus chilensis*	3	1.1	0.4	0.0	1.7
*Turdus falcklandii*	3	0.3	0.2	0.2	0.5
*Tyto alba*	17	0.1	0.1	0.0	0.5
*Vanellus chilensis*	6	0.0	0.0	0.0	0.0
*Zenaida auriculata auriculata*	4	0.4	0.2	0.0	0.7

**Table 3.** Intrageneric K2P genetic distances. Genetic distances within species were excluded.

Genera	Number of taxa	Genetic distances (%)			
		Mean	SE	Minimum	Maximum
* Attagis *	2	4.3	0.0	4.3	4.3
* Charadrius *	5	16.1	1.1	1.9	24.3
* Haematopus *	2	4.3	0.0	4.3	4.3
* Larus *	2	1.3	0.1	1.2	1.4
* Leucophaeus *	2	3.2	0.0	3.2	3.2
* Phalacrocorax *	3	8.5	2.4	3.8	11.4
* Phrygilus *	7	14.3	0.1	2.4	19.4
* Pygoscelis *	2	8.4	0.0	8.4	8.4
* Spheniscus *	2	2.2	0.3	1.9	2.4
* Vanellus *	2	7.7	0.0	7.7	7.7

**Figure 4.**
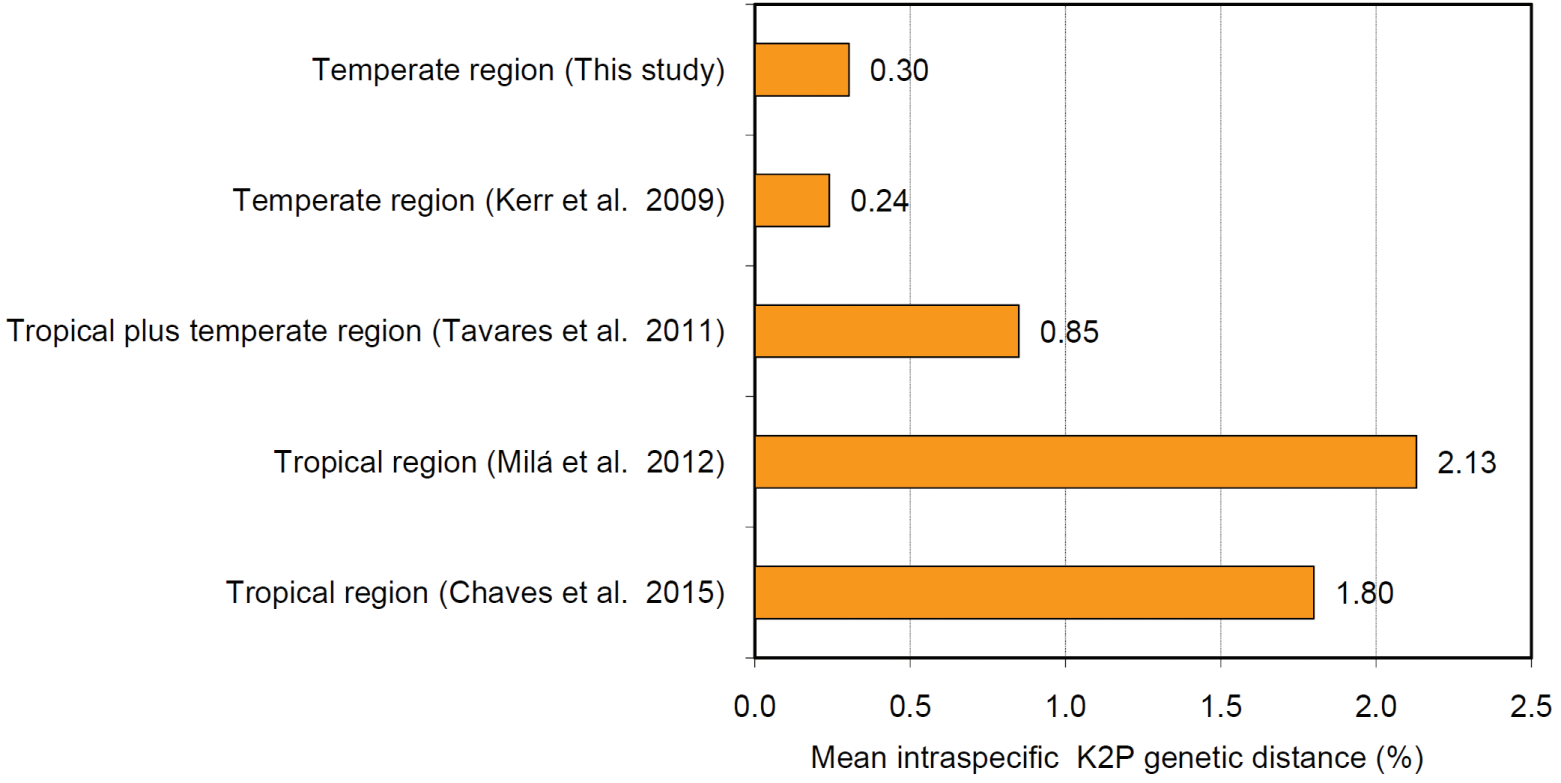
Histogram showing the mean intraspecific genetic distances in bird species from temperate and tropical areas of South America, reported by different research groups.

### Comparisons with previous studies on Neotropical birds

The mean intraspecific divergence in our study (0.3%) was ca. one order of magnitude lower than values reported for bird species in tropical regions of northern South America (1.8 and 2.13%), reported by Chaves et al. (2015) and Milá et al. (2012), respectively. The mean intraspecific divergence in our study was also approximately one-third lower than the value reported by Tavares et al. (2011) (0.9%) for birds distributed throughout South America, including temperate areas of Argentina. However, as shown in Figure 4 our result was similar to the value reported for birds in temperate regions of South America, particularly from Argentina (0.24%) (Kerr et al. 2009). Moreover, our analysis revealed that only 7% of the intraspecific K2P distances exceeded 1.5% K2P, similar to reported findings for Argentine birds, in which 5.4% of species analyzed had a maximum intraspecific distance above 1.5% (Kerr et al. 2009). However, the proportion of species with intraspecific divergence above 1.5% recorded in this study was lower than that reported for birds distributed throughout South America (18.6%) (Tavares et al. 2011).

## Discussion

The mean intraspecific distances recorded in this analysis of Chilean birds (0.3%, based on 43 species) are largely consistent with other reported values for birds in temperate regions of South America, particularly Argentina (0.24%) (Kerr et al. 2009). However, this value was approximately one order of magnitude lower than those found for bird species in tropical regions of northern South America, such as in Brazil (1.8%) (Chaves et al. 2015) and in Ecuador and French Guiana (2.13 %) (Milá et al. 2012). Thus, our results, along with those of Kerr et al. (2009), indicate that birds in temperate regions of South America show less intraspecific genetic variation than birds from tropical regions of the continent. As in other studies, in our case the COI sequence was highly accurate for species-level identification of birds (94.7%), due to a marked barcoding gap. This figure is consistent with previous data indicating that this mitochondrial DNA marker can typically identify 93% or more of the bird species distributed throughout the world, including birds in South America and the northern hemisphere (Barreira et al. 2016). This level of resolution represents a good performance for a single genetic marker. Therefore, the COI sequence may provide a cost-effective tool for screening of biodiversity.

DNA barcoding studies of birds from temperate regions of South America have reported that a relatively small number of species show deep divergence. For instance, Kerr et al. (2009) reported that only 21 of 389 species from Argentina (5.4% of the species studied) showed a maximum intraspecific distance above 1.5%. On the other hand, bird species from tropical regions of South America show substantial genetic divergence. For example, Chaves et al. (2015) reported that 11.6% of Brazilian species had an intraspecific distance above 8.1%. Milá et al. (2012) observed that 75% of species from northern South America had a mean intraspecific divergence above 1%, and more than 50% of species had intraspecific lineage distances above 3%. In our case, few of the species studied (7%) showed a mean intraspecific distance higher than 1.5%. Given that divergence analyses may be affected by various factors such as incomplete or geographically restricted sampling (Meyer and Paulay 2005), further analysis is needed to corroborate our results. Nevertheless, our data are consistent with a previous report on birds from temperate regions of South America (Kerr et al. 2009), supporting the notion that bird species in these regions show low levels of genetic structure and divergence as compared to those from tropical regions. Previous COI studies of Chilean birds that focused on phylogenetic relationships also have also produced evidence of limited intraspecific divergence. For example, three species from different orders exhibited only slight intraspecific genetic divergence, such as *Cyanoliseus patagonus* (Masello et al. 2011), *Aphrastura masafuerae* (González 2014), and *Tyto alba* (Colihueque et al. 2015). Studies based on different molecular markers (ISSR markers) have found similar patterns; *Aphrastura spinicauda*, for instance, showed low levels of genetic diversity among populations distributed across the country (González and Wink 2010).

The limited numbers of bird species with deep divergence in temperate regions of South America may reflect distinct a pattern of regional biodiversity in comparison with tropical avifauna. Alternatively, as noted by Weir and Schluter (2007), the divergence pattern of birds distributed throughout the Americas may reflect a general latitudinal gradient in species diversity. Under this scenario, it would be expected that birds throughout the continent at higher latitudes (toward the poles) would tend to show lower levels of intraspecific divergence than those at lower latitudes (toward the equator). Factors involved in this divergence pattern may include different extinction and speciation rates in the two regions (Weir and Schluter 2007) and greater intraspecific genetic variation in tropical regions (Smith et al. 2017). Thus, the low level of genetic divergence recorded in our study could be related to a general pattern for birds that inhabit the temperate regions of South America. However, it should also be noted that southern South America (above 38°S) was affected by strong glaciation processes in the Quaternary (Hulton et al. 2002). The occurrence of these processes is thought to have impacted various species in southern Chile, including the avifauna (Vuilleumier 1991), by fragmenting their geographical distribution (Villagrán 1990). Therefore, the incidence of local phenomenon related to such climatic fluctuations may also be involved. During glaciation, species survived in refuges and then recolonized sites after resolution of glacial events. As a result, genetic drift, founder effect, or selection may have modified the genetic structure of many species. In the boreal avifauna, the literature suggests that repeated Pleistocene glaciations events may have produced a rapid rate of diversification (Weir and Schluter 2004). Although little is known about the glaciation process associated with the genetic variation of the Chilean avifauna, recent studies suggest that glaciation impacted the genetic structure of specific bird species such as ovenbirds (González and Wink 2010). This notion is based on the finding that within this species, populations currently inhabiting paleorefuge sites show greater genetic variation than populations located in regions that were covered by ice sheets during the Last Glacial Maximum 21,000–14,000 years ago (González and Wink 2010). In addition, the varied genetic patterns of *Phrygilus* species are also consistent with the environmental history of southern South America, including vicariant events and climate changes (Campagna et al. 2011; Álvarez-Varas et al. 2015). Further sampling from a wider latitudinal range will be necessary to produce a more complete view of the genetic structuring and divergence pattern of the bird species analyzed in this work, especially those that include a southern or Patagonian distribution. However, the low levels of genetic variation observed in some species with Patagonian distributions, such as *Vanellus chilensis* and *Charadrius alexandrinus* (currently known as *Charadrius nivosus*) would reveal a genetic structure shaped by glaciation. This conclusion is concordant with the evolutionary history of several Patagonian bird species, whose speciation processes were closely associated with Pleistocene glaciations (Vuilleumier 1991).

A practical utility of DNA barcoding lies in the use of divergence values as a preliminary screening of taxonomic diversity, for example, to screen for within-species divergence. Future work can follow up by examining unusual cases, especially species that show deep divergence. This approach may be useful in understanding the genetic structure of *Curaeus curaeus*, *Phrygilus gayi* and *Sephanoides sephanoides*, as these two species showed the largest intraspecific distances among the species analyzed (above 1.3%). A possible interpretation of this result is that these distances reflect a species complex. In fact, the maximum likelihood tree indicated that *P. gayi* individuals formed at least 3 clusters rather than a cohesive unit, suggestive of different lineages. Álvarez-Varas et al. (2015) have also noted this type of divergence pattern in this species, characterized by genetic clusters associated with different distribution ranges among northern Chilean populations (lowlands vs. Altiplano or highlands). Deep intraspecific divergence in birds may be attributable to the dispersal capacity of the species and/or the presence of barriers to gene flow and environmental heterogeneity. In the case of *P. gayi*, the available evidence indicates that its genetic structure appears to be attributable to ecological factors and a limited dispersal capacity of this species than to geographical factors *per se*, as compared to other species of *Phrygilus* (Álvarez-Varas et al. 2015). For *S. sephanoides*, our finding contrasts with data from a phylogenetic study of the species based on a different set of molecular markers (*Cyt b* and *ND2*) (Roy et al. 1998), which reported non-significant genetic differentiation, estimated as a homogeneity of haplotypes, among north-central and Juan Fernández Islands populations. The misidentification of specimens of this species could be an explanation of for this strong genetic divergence. However, given that samples analyzed showed the typical diagnostic characteristics related to specimen size, feather coloration pattern and other features, this is unlikely. For example, both sequenced specimens show similar feather coloration (upper head and back of metallic green and whitish belly with iridescent green at flank) and body length (ca. 10 cm), which was concordant with the diagnostic criteria reported in ornithology guides of Chile (Jaramillo 2014; Couve et al. 2016). This set of characteristics yields a well-differentiated phenotype as compared to other sympatric species of hummingbirds, such as *Patagona gigas* (Jaramillo 2014). Therefore, it would be difficult to confuse this species with *S. sephanoides.* In sum, there is little doubt as to the conspecificity of the samples. Further analyses with new samples will help to confirm the marked level of divergence observed in *S. sephanoides.* However, given that the individuals analyzed were sampled from distant locations of the country (central and southern Chile, separated by a distance of ca. 800 km), it seems likely that the high level of divergence found in *S. sephanoides* may be related to isolation-by-distance.

The finding that the greatest intrageneric divergence was found in *Charadrius* (16.1%) and *Phrygilus* (14.3%) is noteworthy. In the case of the *Phrygilus* genus this divergence pattern has been interpreted in the context of a marked phylogeographic structure, which is associated with broad altitudinal and latitudinal distributions of species across the Andean mountains (Campagna et al. 2011; Álvarez-Varas et al. 2015). For instance, some species of this group, such as *P. alaudinus*, *P. atriceps* and *P. unicolor*, show a genetic differentiation mediated by allopatric mechanisms in response to specific geographic barriers. In contrast, some genera studied in this work showed low levels of genetic divergence, such as *Larus* (1.3%) and *Spheniscus* (2.2%). Although there is no genetic data for the *Larus* genus in Chile, our results are consistent with data reported for other Laridae species in the northern hemisphere, which show scarce genetic divergence. In fact, the lack of genetic differentiation within this group, reflected in a high sequence similarity (99.8%), gives rise to overlapping barcode clusters with one or more related species (Johnsen et al. 2010). In the case of *Spheniscus*, the evidence obtained in Chile indicate that this genus exhibit lower genetic divergence among species than other penguin genus (e.g., *Pygoscelis*, ca. three fold less variation), based on the analysis of complete mtDNA genomes of a small sample size (Ramos et al. 2018). Future studies in *Spheniscus* with more comprehensive sample sizes will be required to better support the interspecific genetic variation registered in this study based on COI sequence.

In conclusion, this study indicates that DNA barcoding with COI markers is highly accurate for identifying Chilean bird species, as the barcode sequence for nearly every species studied was markedly distinct from that of any other species. Our analysis identified significant interspecific divergence, roughly two orders of magnitude higher than the intraspecific values observed, reflecting a clear barcode gap. In addition, most of the species analyzed showed low intraspecific divergence. This pattern is consistent with data for birds from other temperate regions of southern South America but contrasts with studies on birds from tropical regions of South America, which often show deep intraspecific divergence. Thus, these data reflect the existence of different evolutionary patterns associated with specific regions within the continent. We hope that this step-by-step effort focused on obtaining and assessing the DNA barcodes of the Chilean avifauna, will be useful for increasing knowledge of national biodiversity. This approach may also facilitate the establishment of a dataset of avian barcodes from Chile, enhancing the scope of local studies.

## Supplementary materials

Supplementary material 1 Figure S1. Photographs of external morphology of bird specimens collected from Chile, with lateral, dorsal, or ventral views of specimens showing plumage color and overall appearance. Data type image Explanation note Data on specimen voucher number with size bar to estimate specimen size are provided. https://binary.pensoft.net/file/508469This dataset is made available under the Open Database License (http://opendatacommons.org/licenses/odbl/1.0/). The Open Database License (ODbL) is a license agreement intended to allow users to freely share, modify, and use this Dataset while maintaining this same freedom for others, provided that the original source and author(s) are credited.Nelson Colihueque, Alberto Gantz, Margarita Parraguez

Supplementary material 2 Tables S1 Data type molecular data Explanation note **Table S1.** List of Chilean birds sequenced in this study for the COI marker, with voucher numbers, collection localities, and with BOLD and GenBank accession numbers. For all specimens, tissue samples from muscle were taken. https://binary.pensoft.net/file/508470This dataset is made available under the Open Database License (http://opendatacommons.org/licenses/odbl/1.0/). The Open Database License (ODbL) is a license agreement intended to allow users to freely share, modify, and use this Dataset while maintaining this same freedom for others, provided that the original source and author(s) are credited.Nelson Colihueque, Alberto Gantz, Margarita Parraguez

Supplementary material 3 Tables S2 Data type molecular data Explanation note **Table S2.** List of Chilean bird COI sequences obtained from BOLD and GenBank databases, with voucher numbers and collection localities. https://binary.pensoft.net/file/508471This dataset is made available under the Open Database License (http://opendatacommons.org/licenses/odbl/1.0/). The Open Database License (ODbL) is a license agreement intended to allow users to freely share, modify, and use this Dataset while maintaining this same freedom for others, provided that the original source and author(s) are credited.Nelson Colihueque, Alberto Gantz, Margarita Parraguez
