# Supplementary figures and images for: Revealing the biodiversity of Chilean birds through the COI barcode approach
Source: Zookeys. 2021 Feb 11;1016:143–61. doi: 10.3897/zookeys.1016.51866 (PMC7892532; doi:10.3897/zookeys.1016.51866)

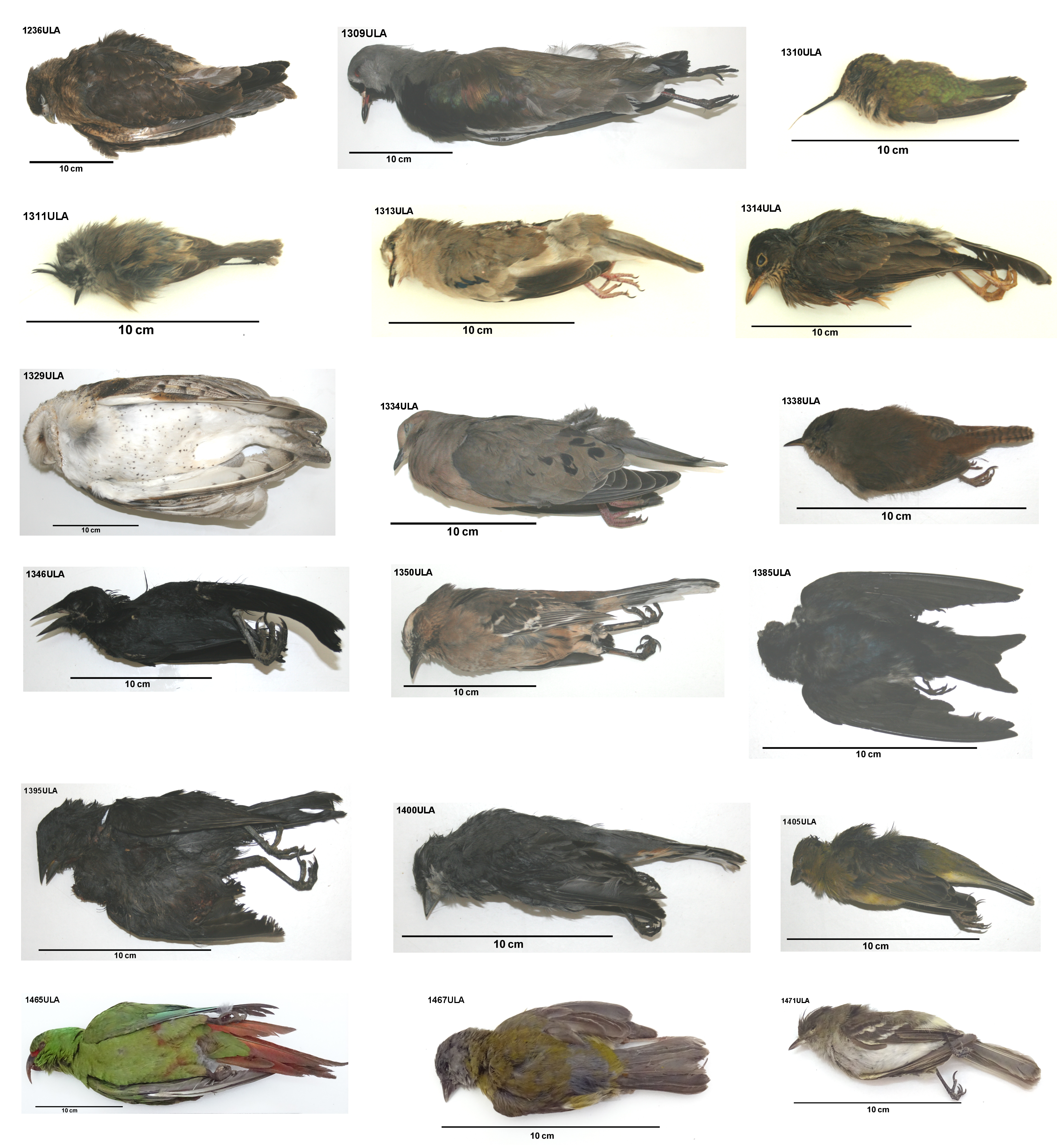

Supplement: Supplementary material 1 — Figure S1. Photographs of external morphology of bird specimens collected from Chile, with lateral, dorsal, or ventral views of specimens showing plumage color and overall appearance. [file zookeys-1016-143-s001.tif]
