# Supplementary material for: Revealing the biodiversity of Chilean birds through the COI barcode approach
Source: Zookeys. 2021 Feb 11;1016:143–61. doi: 10.3897/zookeys.1016.51866 (PMC7892532; doi:10.3897/zookeys.1016.51866)
Supplement: Supplementary material 2 — Tables S1 [file zookeys-1016-143-s002.doc]

| **Supplementary Table 1**. List of Chilean birds sequenced in this study for the COI marker, with voucher numbers, collection localities and with BOLD and GenBank accession numbers. For all specimens tissue samples from muscle were taken. | | | | | | | |
| --- | --- | --- | --- | --- | --- | --- | --- |
| Family | Species or subspecies | Common name | Voucher numbers # | Locality  (Province) | Coordinates |  (Lat., Long.) | Accession numbers | |
|  |  |  |  |  |  | BOLD | GENBANK |
| Accipitridae | *Parabuteo unicinctus* | Harris’ Hawk | 1278ULA | Osorno | 40.48467S, 73.03942W | GBIR12008-19 | MG263847 |
|  | *Megaceryle torquata stellata*† | Ringed Kingfisher (Patagonian) | 1201ULA | Valdivia | 39.813449S, 72.852719W | GBIR11863-19 | MG263840 |
| Anatidae | *Anas georgica* | Yellow-billed Pintail | 1167ULA | Osorno | NA | GBIR10866-19 | MG263832 |
|  | *Anas georgica* | Yellow-billed Pintail | 1215ULA | Cautin | 39.164554S, 72.605412W | GBIR10867-19 | MG263833 |
| Charadriidae | *Vanellus chilensis* | Southern Lapwing | 1245ULA | Osorno | 40.60538S, 72.83562W | GBIR12382-19 | MG263866 |
|  | *Vanellus chilensis* | Southern Lapwing | 1309ULA | Osorno | 40.58440S, 73.09190W | GBIR12381-19 | MG263867 |
|  | *Vanellus chilensis* | Southern Lapwing | 1316ULA | Osorno | 40.59444S, 73.07328W | GBIR12380-19 | MG263870 |
| Columbidae | *Columbina picui* | Picui Ground-Dove | 1313ULA | Cachapoal | 34.2717S, 71.2968W | GBIR11079-19 | MG263868 |
|  | *Zenaida auriculata auriculata* | Eared Dove | 1334ULA | Osorno | 40.5624S, 73.1123W | NA | MN986932 |
|  | *Zenaida auriculata auriculata* | Eared Dove | 1469ULA | Osorno | 40.6031S, 72.8815W | NA | MN986949 |
|  | *Zenaida auriculata auriculata* | Eared Dove | 1470ULA | Osorno | 40.5983S, 72.9818W | NA | MN986950 |
|  | *Zenaida auriculata auriculata* | Eared Dove | 1391ULA | Ranco | 40.3578S, 72.9991W | NA | MN986940 |
| Falconidae | *Falco sparverius* | American Kestrel | 1165ULA | Osorno | NA | GBIR11636-19 | MG263838 |
|  | *Milvago chimango* | Chimango Caracara | 1216ULA | Ranco | 40.332431S, 72.557508W | GBIR11885-19 | MG263841 |
|  | *Milvago chimango* | Chimango Caracara | 1217ULA | Ñuble | 36.658581S, 72.236268W | GBIR11884-19 | MG263842 |
|  | *Milvago chimango* | Chimango Caracara | 1236ULA | Osorno | 40.62770S, 73.07106W | GBIR11881-19 | MG263843 |
|  | *Milvago chimango* | Chimango Caracara | 1237ULA | Osorno | 40.58674S, 73.08755W | GBIR11882-19 | MG263844 |
|  | *Milvago chimango* | Chimango Caracara | 1249ULA | Osorno | 40.60187S, 72.85486W | GBIR11883-19 | MG263845 |
| Fringillidae | *Spinus barbatus¶* | Black-chinned Siskin | 1354ULA | Osorno | 40.5919S, 73.1678W | NA | MN986936 |
|  | *Spinus barbatus¶* | Black-chinned Siskin | 1347ULA | Cachapoal | 34.2717S, 71.2968W | NA | MN986935 |
|  | *Spinus barbatus¶* | Black-chinned Siskin | 1397ULA | Cachapoal | 34.2717S, 71.2968W | NA | MN986953 |
|  | *Spinus barbatus¶* | Black-chinned Siskin | 1460ULA | Cachapoal | 34.2717S, 71.2968W | NA | MN986954 |
| Furnariidae | *Leptasthenura aegithaloides* | Plain-mantled Tit-Spinetail | 1318ULA | Osorno | 40.91691S, 73.14735W | GBIR11858-19 | MG263839 |
| Hirundinidae | *Tachycineta meyeni* | Chilean Swallow | 1388ULA | Osorno | 40.5919S, 73.1678W | GBIR11636-19 | MN986938 |
|  | *Pygochelidon cyanoleuca patagonica* | Blue-and-White Swallow (Patagonica) | 1385ULA | Osorno | 40.5777S, 73.1328W | NA | MN986937 |
| Icteridae | *Curaeus curaeus* | Austral Blackbird | 1346ULA | Cachapoal | 34.2717S, 71.2968W | NA | MN986934 |
|  | *Molothrus bonariensis* | Shiny Cowbird | 1395ULA | Cachapoal | 34.2918S, 71.3097W | NA | MN986942 |
|  | *Molothrus bonariensis* | Shiny Cowbird | 1506ULA | Osorno | 40.5919S, 73.1678W | NA | MN986958 |
| Mimidae | *Mimus thenca* | Chilean Mockingbird | 1350ULA | Osorno | 40.59098S, 73.16869W | GBIR11889-19 | MG263846 |
|  | *Mimus thenca* | Chilean Mockingbird | 1401ULA | Cachapoal | 34.2717S, 71.2968W | NA | MN986944 |
| Odontophoridae | *Callipepla californica* | California Quail | 1468ULA | Cachapoal | 34.2717S, 71.2968W | NA | MN986948 |
| Picidae | *Colaptes pitius* | Chilean Flicker | 1161ULA | Osorno | NA | GBIR11073-19 | MG263835 |
| Psittacidae | *Enicognathus leptorhynchus* | Slender-billed Parakeet | 1160ULA | Osorno | NA | GBIR11296-19 | MG263836 |
|  | *Enicognathus leptorhynchus* | Slender-billed Parakeet | 1295ULA | Osorno | 40.61399S, 72.80733W | GBIR11295-19 | MG263837 |
|  | *Enicognathus leptorhynchus* | Slender-billed Parakeet | 1465ULA | Osorno | 40.7285S, 73.1690W | NA | MN986955 |
|  | *Enicognathus leptorhynchus* | Slender-billed Parakeet | 1466ULA | Osorno | 40.7285S, 73.1690W | NA | MN986956 |
| Rhinocryptidae | *Scytalopus magellanicus* | Magellanic Tapaculo | 1392ULA | Osorno | 40.5919S, 73.1678W | NA | MN986941 |
| Strigidae | *Bubo virginianus magellanicus*‡ | Great Horned Owl (Magellanic) | 1283ULA | Cachapoal | 34.2717S, 71.2968W | GBIR10993-19 | MG263834 |
|  | *Strix rufipes* | Rufous-legged Owl | 1235ULA | Osorno | 40.61205S, 73.09067W | GBIR12309-19 | MG263850 |
|  | *Strix rufipes* | Rufous-legged Owl | 1320ULA | Osorno | NA | GBIR12308-19 | MG263851 |
|  | *Glaucidium nana*& | Austral Pygmy-Owl | 1403ULA | Osorno | 40.7301S, 72.4655W | NA | MN986945 |
| Thraupidae | *Phrygilus patagonicus* | Patagonian Sierra-Finch | 1467ULA | Osorno | 40.6148S, 72.8078 | NA | MN986947 |
|  | *Sicalis luteola* | Grassland Yellow-Finch (Grassland) | 1405ULA | Osorno | 40.5919S, 73.1678W | NA | MN986946 |
|  | *Diuca diuca* | Common Diuca-Finch | 1400ULA | Cachapoal | 34.2717S, 71.2968W | NA | MN986943 |
| Threskiornithidae | *Theristicus melanopis* | Black-faced Ibis | 1194ULA | Osorno | 40.65511S, 73.11928W | GBIR12323-19 | MG263852 |
|  | *Theristicus melanopis* | Black-faced Ibis | 1195ULA | Osorno | 40.60120S, 73.05950W | GBIR12324-19 | MG263853 |
|  | *Theristicus melanopis* | Black-faced Ibis | 1200ULA | Osorno | 40.60233S, 72.86259W | GBIR12321-19 | MG263854 |
|  | *Theristicus melanopis* | Black-faced Ibis | 1214ULA | Osorno | 40.60643S, 73.02569W | GBIR12322-19 | MG263855 |
| Trochilidae | *Sephanoides sephanoides* | Green-backed Firecrown | 1163ULA | Osorno | NA | GBIR12276-19 | MG263848 |
|  | *Sephanoides sephanoides* | Green-backed Firecrown | 1310ULA | Cachapoal | 34.2717S, 71.2968W | GBIR12277-19 | MG263849 |
| Troglodytidae | *Troglodytes musculus chilensis*§ | House Wren (Southern) | 1312ULA | Cachapoal | 34.2717S, 71.2968W | GBIR12358-19 | MG263856 |
|  | *Troglodytes musculus chilensis*§ | House Wren (Southern) | 1338ULA | Osorno | 40.58813S, 73.08889W | GBIR12357-19 | MG263869 |
|  | *Troglodytes musculus chilensis§* | House Wren (Southern) | 1473ULA | Osorno | 40.5919S, 73.1678W | NA | MN986952 |
| Turdidae | *Turdus falcklandii* | Austral Thrush | 1164ULA | Osorno | NA | GBIR12367-19 | MG263857 |
|  | *Turdus falcklandii* | Austral Thrush | 1314ULA | Cachapoal | 34.2717S, 71.2968W | GBIR12366-19 | MG263858 |
|  | *Turdus falcklandii* | Austral Thrush | 1507ULA | Cachapoal | 34.2717S, 71.2968W | NA | MN986959 |
| Tyrannidae | *Anairetes parulus* | Tufted Tit-Tyrant | 1311ULA | Cachapoal | 34.2717S, 71.2968W | GBIR10865-19 | MG263831 |
|  | *Elaenia albiceps chilensis* | White-crested Elaenia (Chilean) | 1471ULA | Osorno | 40.6142S, 72.8074W | NA | MN986951 |
|  | *Elaenia albiceps chilensis* | White-crested Elaenia (Chilean) | 1339ULA | Valdivia | 40.0819S, 72.8726W | NA | MN986933 |
|  | *Elaenia albiceps chilensis* | White-crested Elaenia (Chilean) | 1389ULA | Osorno | 40.5910S, 72.9410W | NA | MN986939 |
|  | *Elaenia albiceps chilensis* | White-crested Elaenia (Chilean) | 1472ULA | Palena | 43.1830S, 71.8665 | NA | MN986957 |
| Tytonidae | *Tyto alba* | Barn Owl (American) | 1277ULA | Ranco | 40.34935S, 72.99702W | GBIR12372-19 | MG263859 |
|  | *Tyto alba* | Barn Owl (American) | 1280ULA | Osorno | 40.77531S, 73.15952W | GBIR12371-19 | MG263860 |
|  | *Tyto alba* | Barn Owl (American) | 1281ULA | Osorno | 40.84398S, 73.17016W | GBIR12373-19 | MG263861 |
|  | *Tyto alba* | Barn Owl (American) | 1282ULA | Osorno | 40.69616S, 73.13282W | GBIR12377-19 | MG263862 |
|  | *Tyto alba* | Barn Owl (American) | 1329ULA | Ranco | 40.23981S, 72.94461W | GBIR12374-19 | MG263863 |
|  | *Tyto alba* | Barn Owl (American) | 1332ULA | Cachapoal | 34.2717S, 71.2968W | GBIR12376-19 | MG263864 |
|  | *Tyto alba* | Barn Owl (American) | 1333ULA | Cachapoal | 34.2717S, 71.2968W | GBIR12375-19 | MG263865 |
| † Synonym with *Ceryle torquata*  ¶ Synonym with *Carduelis barbata*  ‡ Synonym with *Bubo magellanicus*  & Synonym with *Glaucidium nanum*  § Synonym with *Troglodytes aedon chilensis*  # Voucher numbers correspond to the specimen number deposited in the bird collection of the Laboratorio de Biología Molecular y Citogenética of the Universidad de Los Lagos (ULA)  | Coordinates are given in decimal degrees | | | | | | | |
