# Supplementary material for: Revealing the biodiversity of Chilean birds through the COI barcode approach
Source: Zookeys. 2021 Feb 11;1016:143–61. doi: 10.3897/zookeys.1016.51866 (PMC7892532; doi:10.3897/zookeys.1016.51866)
Supplement: Supplementary material 3 — Tables S2 [file zookeys-1016-143-s003.doc]

| **Supplementary Table 2**. List of Chilean bird COI sequences obtained from BOLD and GENBANK databases, with voucher numbers and collection localities. | | | | | | | |
| --- | --- | --- | --- | --- | --- | --- | --- |
| Family | Species | Common name | Voucher number†† | Locality  (Province) | Coordinates  (Lat., Long.) | Accession numbers | |
|  |  |  |  |  |  | BOLD | GENBANK |
| Anatidae | *Chloephaga rubidiceps* | Ruddy-headed Goose | ROM | Magallanes | 53.1S, 70.54W | BROMB190-06 | JN801561 |
|  | *Chloephaga rubidiceps* | Ruddy-headed Goose | ROM | Magallanes | 53.1S, 70.54W | BROMB849-07 | JN801562 |
| Burhinidae | *Burhinus superciliaris* | Peruvian Thick-knee | ROM | Tarapacá | 18.13S, 69.35W | BROM211-06 | NA |
|  | *Burhinus superciliaris* | Peruvian Thick-knee | 159572 ROM | Tarapacá | 18.13S, 69.35W | BROM212-06 | NA |
| Charadriidae | *Charadrius alexandrinus* | Kentish Plover | 158364 ROM | Magallanes | 52.3S, 70.32W | BROM219-06 | NA |
|  | *Charadrius alexandrinus* | Kentish Plover | 158365 ROM | Magallanes | 52.3S, 70.32W | BROM220-06 | NA |
|  | *Charadrius alexandrinus* | Kentish Plover | 158370 ROM | Valparaíso | 33.38S, 71.39W | BROM725-07 | NA |
|  | *Charadrius alticola* | Puna Plover | 159575 | Tarapacá | 18.13S, 69.35W | BROM480-07 | NA |
|  | *Charadrius alticola* | Puna Plover | 159576 | Tarapacá | 18.13S, 69.35W | BROM481-07 | NA |
|  | *Charadrius collaris* | Collared Plover | 158366 | Valparaíso | 33.38S, 71.39W | BROM484-07 | NA |
|  | *Charadrius falklandicus* | Two-banded Plover | 158350 ROM | Magallanes | 53.1S, 70.56W | BROM676-07 | NA |
|  | *Charadrius falklandicus* | Two-banded Plover | 158351 ROM | Magallanes | 53.1S, 70.56W | BROM677-07 | NA |
|  | *Charadrius modestus* | Rufous-chested Dotterel | 158361 ROM | Magallanes | 52.3S, 70.32W | BROM738-07 | NA |
|  | *Charadrius modestus* | Rufous-chested Dotterel | 158352 ROM | Magallanes | 53.1S, 70.56W | BROM685-07 | NA |
|  | *Oreopholus ruficollis* | Tawny-throated Dotterel | 158359 ROM | Magallanes | 52.3S, 70.32W | BROM218-06 | NA |
|  | *Oreopholus ruficollis* | Tawny-throated Dotterel | 158360 ROM | Magallanes | 52.3573S, 70.063W | BROM227-06 | NA |
|  | *Phegornis mitchellii* | Diademed Sandpiper-Plover | ROM | Magallanes | 55.2478S, 68.5009W | BROM438-06 | NA |
|  | *Phegornis mitchellii* | Diademed Sandpiper-Plover | ROM | Copiapó | 27.7167S, 70.2W | BROM439-06 | NA |
|  | *Vanellus chilensis* | Southern Lapwing | 158358 ROM | Magallanes | 53.1S, 70.56W | BROM469-06 | NA |
|  | *Vanellus chilensis* | Southern Lapwing | 158377 ROM | Santiago | 33.27S, 70.4W | BROM468-06 | NA |
|  | *Vanellus chilensis* | Southern Lapwing | 158367 ROM | Valparaíso | 33.38S, 71.39W | BROM870-07 | NA |
|  | *Vanellus resplendens* | Andean Lapwing | 159574 ROM | Tarapacá | 18.13S, 69.35W | BROM590-07 | NA |
| Furnariinae | *Aphrastura masafuerae* | Masafuera Rayadito | NA | Valparaíso‡‡ | NA | GBIR7867-19 | JQ739455 |
|  | *Aphrastura masafuerae* | Masafuera Rayadito | NA | Valparaíso‡‡ | NA | GBIR7866-19 | JQ739454 |
| Haematopodidae | *Haematopus leucopodus* | Magellanic Oystercatcher | 158356 ROM | Magallanes | 53.1S, 70.56W | ROMC022-06 | NA |
|  | *Haematopus leucopodus* | Magellanic Oystercatcher | 158357 ROM | Magallanes | 53.1S, 70.56W | ROMC023-06 | NA |
|  | *Haematopus palliatus* | American Oystercatcher | 158368 ROM | Valparaíso | 33.38S, 71.39W | ROMC054-06 | NA |
|  | *Haematopus palliatus* | American Oystercatcher | 158369 ROM | Valparaíso | 33.38S, 71.39W | ROMC055-06 | NA |
| Hydrobatidae | *Oceanites oceanicus* | Wilson's Storm-Petrel | DOT3175 AMNH | Valparaíso | 33.6S, 71.6W | CDAMH059-05 | DQ433049 |
|  | *Oceanites oceanicus* | Wilson's Storm-Petrel | DOT3176 AMNH | Valparaíso | 33.6S, 71.6W | CDAMH060-05 | DQ433048 |
| Icteridae | *Curaeus curaeus* | Austral Blackbird | AMNH 826156 | Magallanes | NA | GBMNA11448-19 | JX516070 |
| Laridae | *Larus belcheri* | Belcher's Gull | 159570 ROM | Tarapacá | 18.3S, 70.2W | BROM591-07 | NA |
|  | *Larus belcheri* | Belcher's Gull | 159571 ROM | Tarapacá | 18.3S, 70.2W | BROM592-07 | NA |
|  | *Larus dominicanus* | Kelp Gull | CWS | Concepción | 36.24S, 73W | KBNA651-04 | JN801322 |
|  | *Chroicocephalus maculipennis*† | Brown-hooded Gull | 158353 ROM | Magallanes | 53.1S, 70.56W | BROM792-07 | NA |
|  | *Chroicocephalus maculipennis*† | Brown-hooded Gull | 158354 ROM | Magallanes | 53.1S, 70.56W | BROM793-07 | NA |
|  | *Leucophaeus modestus*‡ | Gray Gull | ROM | Tarapacá | 18.3S, 70.2W | BROM797-07 | NA |
|  | *Leucophaeus modestus*‡ | Gray Gull | ROM | Tarapacá | 18.3S, 70.2W | BROM798-07 | NA |
|  | *Leucophaeus modestus*‡ | Gray Gull | 158371 ROM | Valparaíso | 33.38S, 71.39W | BROM799-07 | NA |
|  | *Leucophaeus modestus*‡ | Gray Gull | 158372 ROM | Valparaíso | 33.38S, 71.39W | BROM800-07 | NA |
|  | *Leucophaeus pipixcan*§ | Franklin's Gull | CWS | Chañaral | 26.14S, 70.4W | KBNA631-04 | DQ433764 |
| Mimidae | *Mimus thenca* | Chilean Mocking-bird | Mthenc2676 | NA | NA | GBIR717-08 | EF484228 |
| Pelecanidae | *Pelecanus thagus* | Peruvian Pelican | 21939 CWS | Chañaral | 26.18 S, 70.40 W | KKBNA070-04 | JN801348 |
| Phalacrocoracidae | *Phalacrocorax magellanicus*| | Magellanic Cormorant | NA | NA | NA | GBIR5548-15 | KM066512 |
|  | *Phalacrocorax atriceps* | Imperial Cormorant | NA | NA | NA | GBIR5669-15 | KM066503 |
|  | *Phalacrocorax gaimardi*¶ | Red-legged Cormorant | NA | NA | NA | GBIR5556-15 | KM066530 |
| Pluvianellidae | *Pluvianellus socialis* | Magellanic Plover | ROM | Tierra del Fuego | 53.1654S, 70.2729W | BROM443-06 | NA |
| Procellariidae | *Ardenna creatopus*# | Pink-footed Shearwater | DOT3129 AMNH | Biobío | 37.25S, 72.30W | CDAMH092-05 | DQ433150 |
| Recurvirostridae | *Recurvirostra andina* | Andean Avocet | 159573 ROM | Tarapacá | 18.13S, 69.35W | BROM213-06 | EU525494 |
|  | *Recurvirostra andina* | Andean Avocet | ROM | Tarapacá | 18.13S, 69.35W | BROM214-06 | EU525495 |
| Scolopacidae | *Limosa haemastica* | Hudsonian Godwit | ROM | Magallanes | 52.264S, 69.324W | BROM267-06 | NA |
|  | *Limosa haemastica* | Hudsonian Godwit | ROM | Magallanes | 52.264S, 69.324W | BROM268-06 | NA |
| Spheniscidae | *Eudyptes chrysocome* | Rockhopper Penguin | TRP2203 | Antártica Chilena | 55.4333S, 67.05W | GBIR11338-19 | MG740062 |
|  | *Eudyptes chrysocome* | Rockhopper Penguin | TRP2210 | Antártica Chilena | 55.4333S, 67.05W | GBIR11339-19 | MG740063 |
|  | *Eudyptes chrysocome* | Rockhopper Penguin | TRP2208 | Antártica Chilena | 55.4333S, 67.05W | GBIR11340-19 | MG740064 |
|  | *Eudyptes chrysocome* | Rockhopper Penguin | TRP2207 | Antártica Chilena | 55.4333S, 67.05W | GBIR11341-19 | MG740065 |
|  | *Eudyptes chrysocome* | Rockhopper Penguin | TRP2206 | Antártica Chilena | 55.4333S, 67.05W | GBIR11342-19 | MG740066 |
|  | *Eudyptes chrysocome* | Rockhopper Penguin | TRP2204 | Antártica Chilena | 55.4333S, 67.05W | GBIR11343-19 | MG740067 |
|  | *Eudyptes chrysocome* | Rockhopper Penguin | TRP2201 | Antártica Chilena | 55.4333S, 67.05W | GBIR11344-19 | MG740068 |
|  | *Eudyptes chrysocome* | Rockhopper Penguin | TRP2209 | Antártica Chilena | 55.4333S, 67.05W | GBIR11345-19 | MG740069 |
|  | *Pygoscelis antarcticus* | Chinstrap Penguin | Isolate 3 PUC | Península Antártica | 63.3166S, 57.90W | GBIR10303-19 | KU356674 |
|  | *Pygoscelis papua* | Gentoo Penguin | Isolate 12 PUC | Península Antártica | 64.8000S, 62.85W | GBMNA11626-19 | KU356677 |
|  |  |  |  |  |  |  |  |
|  | *Spheniscus humboldti* | Humboldt's Penguin | Isolate 6 PUC | Petorca | 32.5833S, 71.45W | GBIR10304-19 | KU361805 |
|  | *Spheniscus magellanicus* | Magellanic penguin | Isolate 4 PUC | Chiloé | 41.9166S, 74.03W | GBIR10305-19 | KU361804 |
|  | *Spheniscus magellanicus* | Magellanic penguin | Isolate 7 PUC | Magallanes | 52.85S, 70.60W | GBIR10306-19 | KU361806 |
| Stercorariidae | *Stercorarius chilensis* | Chilean Skua | 158363 ROM | Magallanes | 53.1S, 73.56W | BROM217-06 | NA |
| Strigidae | *Asio flammeus* | Short-eared Owl | 1188 ULA | Osorno | 40.6014S, 72.8888W | GBMIN133522-17 | KM377628 |
| Thinocoridae | *Attagis gayi* | Rufous-bellied Seedsnipe | 159569 ROM | Tarapacá | 18.13S, 69.35W | BROM382-06 | NA |
|  | *Attagis gayi* | Rufous-bellied Seedsnipe | ROM | Tarapacá | 18.13S, 69.35W | BROM383-06 | KF009515 |
|  | *Attagis gayi* | Rufous-bellied Seedsnipe | ROM | Tarapacá | 18.13S, 69.35W | BROM384-06 | NA |
|  | *Attagis malouinus* | White-bellied Seedsnipe | ROM | Magallanes | 52.28S, 70.33W | BROM712-07 | KF009516 |
|  | *Thinocorus orbignyianus* | Gray-breasted Seedsnipe | 158373 ROM | Santiago | 33.3S, 70.3W | BROM532-07 | NA |
|  | *Thinocorus orbignyianus* | Gray-breasted Seedsnipe | 158375 ROM | Santiago | 33.3S, 70.3W | BROM585-07 | NA |
|  | *Thinocorus orbignyianus* | Gray-breasted Seedsnipe | 158374 ROM | Santiago | 33.3S, 70.3W | BROM859-07 | NA |
|  | *Thinocorus orbignyianus* | Gray-breasted Seedsnipe | 159578 | Tarapacá | 18.13S, 69.35W | BROM533-07 | NA |
|  | *Thinocorus orbignyianus* | Gray-breasted Seedsnipe | 159577 ROM | Tarapacá | 18.13S, 69.35W | BROM589-07 | KF147200 |
|  | *Thinocorus orbignyianus* | Gray-breasted Seedsnipe | ROM | Tarapacá | 18.13S, 69.35W | BROM858-07 | NA |
| Thraupidae | *Phrygilus alaudinus* | Band-tailed Sierra-Finch | PA3 | Huasco | 28.02S, 71.50W | GBIR6784-15 | KP015143 |
|  | *Phrygilus alaudinus* | Band-tailed Sierra-Finch | PA56 | Huasco | 28.02S, 71.50W | GBIR6785-15 | KP015144 |
|  | *Phrygilus alaudinus* | Band-tailed Sierra-Finch | PA82 | Huasco | 28.02S, 71.50W | GBIR6786-15 | KP015145 |
|  | *Phrygilus alaudinus* | Band-tailed Sierra-Finch | PA48 | Huasco | 28.02S, 71.50W | GBIR6787-15 | KP015146 |
|  | *Phrygilus alaudinus* | Band-tailed Sierra-Finch | PA46 | Huasco | 28.02S, 71.50W | GBIR6788-15 | KP015147 |
|  | *Phrygilus alaudinus* | Band-tailed Sierra-Finch | PA52 | Huasco | 28.02S, 71.50W | GBIR6789-15 | KP015148 |
|  | *Phrygilus alaudinus* | Band-tailed Sierra-Finch | PA45 | Huasco | 28.02S, 71.50W | GBIR6790-15 | KP015149 |
|  | *Phrygilus alaudinus* | Band-tailed Sierra-Finch | PA42 | Huasco | 28.02S, 71.50W | GBIR6791-15 | KP015150 |
|  | *Phrygilus alaudinus* | Band-tailed Sierra-Finch | PA61 | Huasco | 28.02S, 71.50W | GBIR6792-15 | KP015151 |
|  | *Phrygilus alaudinus* | Band-tailed Sierra-Finch | PA6 | Huasco | 28.02S, 71.50W | GBIR6793-15 | KP015152 |
|  | *Phrygilus alaudinus* | Band-tailed Sierra-Finch | PA64 | Huasco | 28.02S, 71.50W | GBIR6794-15 | KP015153 |
|  | *Phrygilus alaudinus* | Band-tailed Sierra-Finch | PA19 | Huasco | 28.02S, 71.50W | GBIR6797-15 | KP015156 |
|  | *Phrygilus alaudinus* | Band-tailed Sierra-Finch | PA26 | Huasco | 28.02S, 71.50W | GBIR6798-15 | KP015157 |
|  | *Phrygilus alaudinus* | Band-tailed Sierra-Finch | PA96 | Limarí | 30.67S, 71.67W | GBIR6798-15 | KP015154 |
|  | *Phrygilus alaudinus* | Band-tailed Sierra-Finch | PA88 | Limarí | 30.67S, 71.67W | GBIR6796-15 | KP015155 |
|  | *Phrygilus atriceps* | Black-hooded Sierra-Finch | PAT1 | Iquique | 19.69S, 69.18W | GBIR6799-15 | KP015158 |
|  | *Phrygilus atriceps* | Black-hooded Sierra-Finch | PAT2 | Iquique | 19.69S, 69.18W | GBIR6800-15 | KP015159 |
|  | *Phrygilus atriceps* | Black-hooded Sierra-Finch | PAT3 | Parinacota | 18.25S, 69.08W | GBIR6801-15 | KP015160 |
|  | *Phrygilus fruticeti* | Mourning Sierra-Finch | PF6 | Limarí | 31.47S, 71.05W | GBIR6802-15 | KP015161 |
|  | *Phrygilus fruticeti* | Mourning Sierra-Finch | PF8 | Limarí | 31.47S, 71.05W | GBIR6803-15 | KP015162 |
|  | *Phrygilus fruticeti* | Mourning Sierra-Finch | PF15 | Limarí | 31.47S, 71.05W | GBIR6804-15 | KP015163 |
|  | *Phrygilus fruticeti* | Mourning Sierra-Finch | PF12 | Limarí | 31.47S, 71.05W | GBIR6806-15 | KP015165 |
|  | *Phrygilus fruticeti* | Mourning Sierra-Finch | PF38 | Parinacota | 18.27S, 69.58W | GBIR6805-15 | KP015164 |
|  | *Phrygilus fruticeti* | Mourning Sierra-Finch | PF39 | Parinacota | 18.25S, 69.60W | GBIR6807-15 | KP015166 |
|  | *Phrygilus fruticeti* | Mourning Sierra-Finch | PF51 | Parinacota | 18.25S, 69.60W | GBIR6808-15 | KP015167 |
|  | *Phrygilus gayi* | Gray-hooded Sierra-Finch | PG4 | Huasco | 28.02S, 71.50W | GBIR6810-15 | KP015169 |
|  | *Phrygilus gayi* | Gray-hooded Sierra-Finch | PG3 | Huasco | 28.02S, 71.50W | GBIR6809-15 | KP015171 |
|  | *Phrygilus gayi* | Gray-hooded Sierra-Finch | PG1 | Limarí | 30.67S, 71.67W | GBIR6811-15 | KP015168 |
|  | *Phrygilus gayi* | Gray-hooded Sierra-Finch | PG269 | Colchagua | 34.96S, 70.43W | GBIR6812-15 | KP015170 |
|  | *Phrygilus gayi* | Gray-hooded Sierra-Finch | PG172 | Colchagua | 34.96S, 70.43W | GBIR6814-15 | KP015173 |
|  | *Phrygilus gayi* | Gray-hooded Sierra-Finch | PG184 | Colchagua | 34.96S, 70.43W | GBIR6813-15 | KP015174 |
|  | *Phrygilus gayi* | Gray-hooded Sierra-Finch | PG230 | Colchagua | 34.96S, 70.43W | GBIR6815-15 | KP015172 |
|  | *Phrygilus plebejus* | Ash-breasted Sierra-Finch | PLE14 | El Loa | 21.22S, 68.27W | GBIR6816-15 | KP015134 |
|  | *Phrygilus plebejus* | Ash-breasted Sierra-Finch | PLE4 | El Loa | 21.22S, 68.27W | GBIR6817-15 | KP015135 |
|  | *Phrygilus plebejus* | Ash-breasted Sierra-Finch | PLE18 | El Loa | 21.22S, 68.27W | GBIR6818-15 | KP015136 |
|  | *Phrygilus plebejus* | Ash-breasted Sierra-Finch | PLE15 | El Loa | 21.22S, 68.27W | GBIR6819-15 | KP015137 |
|  | *Phrygilus plebejus* | Ash-breasted Sierra-Finch | PLE2 | El Loa | 21.22S, 68.27W | GBIR6820-15 | KP015138 |
|  | *Phrygilus plebejus* | Ash-breasted Sierra-Finch | PLE13 | El Loa | 21.22S, 68.27W | GBIR6821-15 | KP015139 |
|  | *Phrygilus plebejus* | Ash-breasted Sierra-Finch | PLE7 | El Loa | 21.22S, 68.27W | GBIR6822-15 | KP015140 |
|  | *Phrygilus unicolor* | Plumbeous Sierra-Finch | PHU1 | Parinacota | 18.25S, 69.08W | GBIR6823-15 | KP015141 |
|  | *Phrygilus unicolor* | Plumbeous Sierra-Finch | PHU2 | Parinacota | 18.25S, 69.08W | GBIR6824-15 | KP015142 |
| Tinamidae | *Tinamotis pentlandii* | Puna Tinamou | ROM | Tarapacá | 18.13S, 69.35W | BROMB209-06 | JN802048 |
| Tytonidae | *Tyto alba* | Barn Owl (American) | 1189ULA | Osorno | 40.6054S, 73.045W | GBMIN133523-17 | KM377629 |
|  | *Tyto alba* | Barn Owl (American) | 1225ULA | Osorno | 40.4129S, 73.0001W | GBMIN133526-17 | KM377632 |
|  | *Tyto alba* | Barn Owl (American) | 1233ULA | Osorno | 40.6929S, 73.1313W | GBMIN133532-17 | KM377638 |
|  | *Tyto alba* | Barn Owl (American) | 1224ULA | Ranco | 40.2975S, 72.9741W | GBMIN133525-17 | KM377631 |
|  | *Tyto alba* | Barn Owl (American) | 1226ULA | Ranco | 40.3578S, 72.9991W | GBMIN133527-17 | KM377633 |
|  | *Tyto alba* | Barn Owl (American) | 1227ULA | Ranco | 40.3065S, 72.9788W | GBMIN133528-17 | KM377634 |
|  | *Tyto alba* | Barn Owl (American) | 1228ULA | Ranco | 40.2998S, 72.9497W | GBMIN133529-17 | KM377635 |
|  | *Tyto alba* | Barn Owl (American) | 1229ULA | Ranco | 40.1843S, 72.9138W | GBMIN133530-17 | KM377636 |
|  | *Tyto alba* | Barn Owl (American) | 1223ULA | Valdivia | 40.0818S, 72.8726W | GBMIN133524-17 | KM377630 |
|  | *Tyto alba* | Barn Owl (American) | 1231ULA | Valdivia | 39.8452S, 72.8112W | GBMIN133531-17 | KM377637 |
| † Synonym with *Larus maculipennis*  ‡ Synonym with *Larus modestus*  § Synonym with *Larus pipixcan*  | Synonym with *Leucocarbo magellanicus*  ¶ Synonym with *Poikilocarbo gaimardi*  & Synonym with *Phalacrocorax bougainvillii*  # Synonym with *Puffinus creatopus*  †† ROM = Royal Ontario Museum, AMNH = American Museum of Natural History, CWS = Canadian Wildlife Service, MACN = Museo Argentino de Ciencias Naturales Bernandino Rivadavia, PUC = Pontificia Universidad Católica de Chile, ULA = Universidad de Los Lagos, NA = Not available  ‡‡ Locality corresponds to Juan Fernández islands | | | | | | | |
